# Cerebrovascular Reactivity and Neurovascular Coupling in Multiple Sclerosis—A Systematic Review

**DOI:** 10.3389/fneur.2022.912828

**Published:** 2022-06-01

**Authors:** Mark B. Vestergaard, Jette L. Frederiksen, Henrik B. W. Larsson, Stig P. Cramer

**Affiliations:** ^1^Functional Imaging Unit, Department of Clinical Physiology and Nuclear Medicine, Copenhagen University Hospital Rigshospitalet, Glostrup, Denmark; ^2^Danish Multiple Sclerosis Center, Department of Neurology, Copenhagen University Hospital Rigshospitalet, Glostrup, Denmark; ^3^Department of Clinical Medicine, Faculty of Health and Medical Science, University of Copenhagen, Copenhagen, Denmark

**Keywords:** cerebral blood flow (CBF), cerebrovascular disease, cerebrovascular hemodynamics, cerebrovascular reactivity (CVR), multiple sclerosis, neurovascular coupling (NVC)

## Abstract

The inflammatory processes observed in the central nervous system in multiple sclerosis (MS) could damage the endothelium of the cerebral vessels and lead to a dysfunctional regulation of vessel tonus and recruitment, potentially impairing cerebrovascular reactivity (CVR) and neurovascular coupling (NVC). Impaired CVR or NVC correlates with declining brain health and potentially plays a causal role in the development of neurodegenerative disease. Therefore, we examined studies on CVR or NVC in MS patients to evaluate the evidence for impaired cerebrovascular function as a contributing disease mechanism in MS. Twenty-three studies were included (12 examined CVR and 11 examined NVC). Six studies found no difference in CVR response between MS patients and healthy controls. Five studies observed reduced CVR in patients. This discrepancy can be because CVR is mainly affected after a long disease duration and therefore is not observed in all patients. All studies used CO_2_ as a vasodilating stimulus. The studies on NVC demonstrated diverse results; hence a conclusion that describes all the published observations is difficult to find. Future studies using quantitative techniques and larger study samples are needed to elucidate the discrepancies in the reported results.

## Introduction

Multiple sclerosis (MS) is characterized by autoimmune inflammation in the central nervous system, which primarily targets myelin and oligodendrocytes, with secondary damage to the axons and potential degeneration of the neuron itself through Wallerian degeneration (1). Activated astrocytes and microglia contribute to the creation of a proinflammatory milieu by releasing cytokines and chemokines, attracting immune cells to the lesion area and allowing the migration of inflammatory cells into the brain parenchyma through a compromised blood–brain barrier (BBB) (2–7). The inflammatory processes also cause damage to the vessel endothelium in the brain, possibly through cytokine-mediated mechanisms or by the production of reactive oxygen species (ROS) (8–10). Endothelium damage can cause cerebrovascular dysfunction and impaired cerebral perfusion modulation, as the endothelium is involved in the regulation of vasomotor tone trough the release of vasodilating agents (11). Dysfunction in endothelially mediated dilation will reduce the responsiveness of the vessels to vasodilating stimuli.

Cerebrovascular dysfunction is observed in Alzheimer's disease (AD) and vascular dementia (VD), and increasing evidence suggests that cerebrovascular dysfunction can be a causal aspect in the development of age-related neurodegenerative diseases (12–14). Both reduced cerebrovascular reactivity (CVR) to vasodilating stimuli such as carbon dioxide (CO_2_) and impaired neurovascular coupling (NVC) are observed in patients with AD or VD (15–21). Reduced CVR also correlates with cognitive performance in elderly human subjects without neurodegenerative disease (22), whereas a healthy lifestyle can maintain a responsive cerebrovascular function with advancing age (23–25). In summary, these studies demonstrate that having healthy cerebrovascular function, which can readily increase cerebral blood flow (CBF) to a region with an increased metabolic demand, is likely an important factor in maintaining a healthy brain, especially considering age-related declines in brain health.

Studies on postmortem brain cells from MS patients have demonstrated hypoxia-like tissue damage in both gray and white matter (26–28), which could be an indication of vessel damage and cerebrovascular dysfunction. Experimental-autoimmune-encephalopathy (EAE) animal models of MS have also demonstrated that tissue hypoxia plays a vital role not only in the formation of demyelinating lesions but also in cortical atrophy and that hyperbaric treatment may dampen this process (29–31). Moreover, whether tissue hypoxia may be an important factor in explaining the highly transient neurological deficits observed in MS remains to be elucidated (31). Overall, these observations have led to speculations on whether tissue hypoxia and impaired cerebrovascular function make important contributions to the pathophysiology of MS, warranting further research from a treatment perspective. If patients with MS have impaired cerebrovascular function, then this could be part of a pathophysiological cascade aggravating brain atrophy. Therefore, examining and understanding how cerebrovascular function is affected in MS is of high clinical importance. Several studies have shown that cerebral hypometabolism and hypoperfusion are present in MS (32–34); however, these studies are not ideal to conclude on the causality between vasophysiology and brain atrophy as a reduced resting metabolism, especially in later stages of MS disease in itself can be a result of brain atrophy. Thus, the cerebrovascular response to a vasodilating stimulus may be a more suitable indicator of vascular dysfunction.

In the present systemic review, we examined the existing literature on CVR and NVC in patients with MS. We investigated whether evidence of impaired cerebrovascular function in MS could be found.

### Examination of Cerebrovascular Reactivity

CBF is highly regulated to maintain adequate perfusion in the brain. Changes in metabolic demand rapidly leads to increased perfusion to the affected brain area. Similarly, changes in systemic factors, such as changes in blood pressure or arterial oxygen saturation, are rapidly counteracted to maintain a stable blood and oxygen supply to the brain (35). Cerebral perfusion is mainly regulated by changes in vascular resistance through the dilation and constriction of the cerebral arteries and capillaries. The dilation and constriction of arteries and arterioles are controlled by vascular smooth muscle cells (VSMCs), and the dilation and constriction of capillaries are controlled by pericytes (36).

CVR is the reactivity of the CBF response to an external isometabolic vasodilating stimulus. The preferred stimulus is exposure to CO_2_, a potent vasodilator of the cerebral arteries and arterioles (37), which is thought to cause minimal to no effect on cerebral metabolism (38, 39). The vasodilating effect of CO_2_ on VSMCs is mediated through the production of nitric oxide (NO) and bicarbonate (${\text{HCO}}_{3}^{-}$) from a decrease in pH (40). Thus, using CO_2_ as a vasodilating agent provides a good measure of VSMC function and the vasomotor capabilities of the cerebral arteries. Therefore, damage to the endothelium from inflammatory processes in patients with MS is therefore hypothesized to cause reduced vasodilation from a CO_2_ stimulus. The typical procedure when examining CVR is to measure CBF at rest and to repeat this measurement during CO_2_ exposure. Exposure to CO_2_ is obtained either by the inhalation of air with added CO_2_ or by breath-hold challenges that increase tissue and blood CO_2_ partial pressure (41–43). CBF is measured by either neuroimaging techniques, such as positron emission tomography (PET) or arterial spin labeling (ASL) magnetic resonance imaging (MRI), or by measuring the blood flow or velocity in the feeding cerebral arteries using transcranial Doppler ultrasound (TCD) or phase contrast mapping (PCM) MRI (42, 44–48).

### Examination of Neurovascular Coupling

The NVC describes the local increase in CBF evoked by neuronal activation. The NVC functions in a highly complex manner and involves multiple intermediate steps. The release of neurotransmitters from neuroactivation, most notably glutamate, leads to the release of vasoactive agents through a feedforward mechanism (49). A major pathway for vasodilation acts through synaptic glutamate by triggering N-methyl-d-aspartate (NMDA) receptors, which in turn mediate the influx of Ca^2+^ into the neurons, where the higher intracellular Ca^2+^ promotes NO synthase (nNOS) and the production of NO (50, 51). NO easily diffuses into the surrounding arteries, causing vasodilation through the generation of cyclic guanosine monophosphate (cGMP) in VSMCs (52). Astrocytes also play an important role in NVC and in the mediation of vasodilation (53–55). The influx of Ca^2+^ in astrocytes from glutamate activation promotes the release of arachidonic acid and prostaglandins, both of which have vasodilating effects. Additionally, the higher Ca^2+^ concentration in astrocyte end-feet will open K^+^ channels, releasing K^+^ to the intercellular space and causing direct vasodilation effects on the surrounding vessels (56). Reductions in the local oxygen pressure also have a marked regulatory effect on the accumulation of vasoactive agents. Reduced oxygen availability promotes astrocytic lactate production and release. Extracellular lactate attenuates the uptake of extracellular prostaglandin E2. Prostaglandin E2 accumulation leads to vasodilation (53). At the capillary level, the dilation of the vessel is controlled by pericytes through pathways similar to those of the VSMC control (36). Endothelial cells of the arterioles propagate the vasodilating signal upstream to the penetrating and pial arteries (57). Thus, the quantification of the NVC reflects the integrated sum of a complex cerebrovascular function with multiple steps aimed at increasing CBF. Reduced NVC in MS patients could arise from a malfunction in one or more of the multiple steps in the neurovascular cascade.

Measuring NVC *in vivo* in humans is challenging. To directly examine NVC, measurements of both the change in neuronal electrical activity and the corresponding change in CBF are needed and should preferably be examined in a quantitative manner. The measurements must also be acquired with a sufficiently fast time resolution to assess the rapid focal change in metabolism and electrical activity from neuroactivation. Ideally, the measurements of neuronal activity and CBF should also be acquired simultaneously. Brain electrical activity can be examined using electroencephalography (EEG) and magnetoencephalography (MEG) techniques (58). By default, these techniques are not quantitative; however, by calculating the spectral power of the measurements, the relative change in neuronal activity can be assessed (59, 60). Instead of measuring electrical activity, some studies instead acquire the change in oxygen usage [cerebral metabolic rate of oxygen (CMRO_2_)] from neuroactivation under the assumption of a strong correlation between electrical activity and energy consumption in neurons (61–63).

Given the difficulties in measuring neuronal activation, many studies only acquire the CBF response as a surrogate marker of NVC and then assume that neuronal electrical activation is constant across the studied population. Perfusion PET imaging can be used to measure the CBF response to neuroactivation by acquiring resting perfusion maps and perfusion maps during neuroactivation (64, 65). PET imaging is considered the gold standard for measuring quantitative CBF maps *in vivo*; however, due to the invasive procedure from the use of radioactive tracers and the need for arterial blood samples for an input function for kinetic modeling (46, 66, 67), PET imaging is rarely used to examine NVC in a research setting. Instead, the non-invasive arterial spin labeling (ASL) MRI technique can be applied to measure changes in CBF from neuronal activation (68). Additionally, by combining ASL with blood-oxygen-level-dependent (BOLD) imaging, a technique referred to as calibrated BOLD (cBOLD), the change in CMRO_2_ from neuroactivation can be calculated (69). Traditional BOLD imaging has also been used to examine the CBF response to activation as an indirect measure of NVC. By using the BOLD imaging, the temporal evolution of the hemodynamic response function (HRF), e.g., time to peak and peak amplitude, of the NVC can be characterized.

TCD has also been used to assess the CBF response to neuroactivation (70). This technique measures the blood velocity in one of the larger cerebral arteries supplying the activated brain region. Typically, the change in blood velocity in the posterior cerebral arteries (PCA) or middle cerebral artery (MCA) during visual activation is used as a marker of the CBF response to neuroactivation. The examination of CBF to neuroactivation by TCD serves as an indirect measurement because the regional change in perfusion in the activated region is not obtained, but only the upstream change in blood flow velocity (CBF_v_) in the large artery supplying the activated brain region is measured. Given that the artery supplies not only the activated region but also other areas of the brain, the measured change in blood velocity is a diluted average of a much larger brain area. When measuring CBF by TCD, the response to visual stimulation results in an increase in the blood velocity through the PCA by ~5–40% and through the MCA by ~0–10% (71–73), in contrast to the much larger focal increase in perfusion in the visual cortex, which is ~50-100% (64, 66, 68, 74). Thus, the examination of the CBF response by TCD is not fully quantifiable.

When assessing NVC, neuroactivation is typically obtained by visual stimulation of the participants, often in the form of a flickering light pattern eliciting a strong neuroactivation in the visual cortex (75).

## Methods

We conducted the review according to the Preferred Reporting Items for Systematic Reviews and Meta-Analysis (PRISMA) Statement (76).

### Search Strategy

The search for articles to include in the systematic review was carried out via PubMed using a combination of free text words and MeSH terms. The initial search was performed on 31st of May 2021. The search was repeated on 30th of November 2021 to include papers published since the initial search.

The following PubMed search string was used:

[“multiple sclerosis”[MeSH Terms] OR “multiple sclerosis, relapsing remitting”[MeSH Terms] OR “progressive multiple sclerosis”[Text Word] OR “multiple sclerosis”[Text Word]) AND (“cerebrovascular reactivity”[Text Word] OR “Vasomotor”[Text Word] OR “vasodilation”[Text Word] OR “vasomotor reactivity”[Text Word] OR “cerebrovascular reserve capacity”[Text Word] OR “Cerebral autoregulation”[Text Word] OR “arterial stiffness“[Text Word] OR “endothelium dysfunction”[Text Word] OR “endothelium damage”[Text Word] OR “endothelium injury”[Text Word] OR “hemodynamic”[Text Word] OR “hemodynamic response function”[Text Word] OR “neurovascular coupling”[Text Word]].

### Eligibility Criteria

Studies investigating CVR or NVC in a quantitative or semiquantitative fashion in MS patients were included in the review. Only original research studies were included. Articles in languages other than English were not included. All papers were initially screened by MBV based on the title and abstract. Papers that passed the initial screening were included in a full-text eligibility assessment. The reference lists of the included papers were reviewed for additional eligible articles.

### Data Extraction

The CVR and NVC results were extracted as the primary output. The techniques used to measure CVR and NVC (MRI, TCD etc.) and the means of vasodilation and neuronal activation (CO_2_ inhalation, breath-holding, visual stimulation etc.) were additionally extracted. Finally, we identified data regarding the participants (i.e., type of MS, disease duration, medication status, current disease status, Expanded Disability Status Scale (EDSS) and additional disease parameters if provided).

The data extracted from the included studies are summarized in Tables 1, 2.

**Table 1:** Summary of studies examining cerebrovascular reactivity in MS.

**Paper**	**Subjects**	**Study comment**	**Modality**	**Stimulus**	**Primary outcome**	**Results**
Uzuner et al. (77)	12 RRMS patients and 11 HC (no information on MS subtype).	Patients were examined three times during exacerbation and after treatment with methylprednisolone.	Blood velocity in MCAs (CBF_v_) by TCD.	15 sec breath holds.	BHI as the maximal percentage change of CBF_v_ during breath hold.	No difference in BHI between MS and HC either during attacks or after treatment.
Marshall et al. (78)	19 MS patients (17 RRMS and 2 SPMS) and 19 HC. EDDS = 2.9 ± 1.5 (range: 1–6). Disease duration = 10.4 ± 7.6 years (range: 2.2–24).	Most patients received second line treatment. Large variation of clinical symptoms.	3T MRI. CBF maps obtained by single TI pCASL sequence.	Inhalation of air with 5% CO_2_.	CVR as the percentage change of CBF normalized to change in P_et_CO_2_ in response to CO_2_ exposure.	Global CVR was reduced in MS compared with HC. CVR correlated negatively with white matter lesion load and gray matter atrophy.
Marshall et al. (79)	28 MS patients (26 RRMS and 2 SPMS) and 28 HC. EDDS = 2.9 ± 1.6 (range: 1 – 6). Disease duration = 9.84 ± 7.7 years (range: 1.3–29).	Patients mostly received second line treatment. Large variation of clinical symptoms.	3T MRI. CBF maps obtained by single TI pCASL sequence.	Inhalation of air with 5% CO_2_.	CVR as the change of CBF normalized to change in P_et_CO_2_ in response to CO_2_ exposure.	Global CVR was reduced in MS compared with HC. CVR correlated negatively with white matter lesion load.
Khorvash et al. (80)	40 MS patients (no information on MS subtype) and 40 migraine patients as control group. No information on EDDS or disease duration.		Blood velocity in MCAs (CBF_v_) by TCD.	30 sec breath holds.	Main flow velocity (MFV) in MCAs and BHI. An exact procedure for calculating BHI was not provided.	MS patients had higher BHI and MFV during breath holding than the migraine HC.
Metzger et al. (81)	33 patients (24 RRMS and 9 progressive MS, not reported whether SPMS or PPMS) and 22 HC. EDDS = 3 ± 2.05. Disease duration = 8 ± 8.1 years.	Patients divided into a cognitive impaired group (*n* = 12) and cognitive normal group (*n* = 21).	3T MRI. BOLD maps measured using a gradient-echo EPI sequence.	Inhalation of air with 8% CO_2_.	Change in BOLD signal (CVR_BOLD_) normalized to the change in P_et_CO_2_ in response to CO_2_ exposure.	CVR_BOLD_ was reduced in cognitively impaired MS patients when compared with cognitive normal patients. No difference between patients and HC.
Krogias et al. (82)	42 patients with MS (27 RRMS and rest were not reported) and 31 HC. EDDS = 2.7 ± 2.1 (range: 1–8). Disease duration = 9.3 ± 8.4 years (range 0.5–34).	No relapses or use of methylprednisolone within 3 months.	Blood velocity in MCAs (CBF_v_) by TCD.	30 sec breath holds.	BHI as percentage change of CBF_v_ at end of breath hold compared to rest.	BHI was in reduced in MS patients compared with HC.
Pelizzari et al. (83)	31 MS (29 RRMS and 2 SPMS) and 25 HC. EDSS [median] = 1.5 (range: 1–7). Disease duration = 8 years (range: 2–36).	No relapses or use of methylprednisolone within 3 months. Most patients received first line treatment.	1.5T MRI. CBF maps measured by multi-TI pCASL sequence.	Inhalation of air with 5% CO_2_.	CVR as percentage change of CBF normalized to the change in P_et_CO_2_ in response to CO_2_ exposure.	No difference in CVR between patients and HC.
Lattanzi et al. (84)	80 patients (48 RRMS and 32 SPMS) and 80 HC. EDSS [median] = 3.5 (range: 2–6). Disease duration = 11 years (range: 7–20).	Patients received mostly second line treatment.	Blood velocity in MCAs (CBF_v_) by TCD.	30 sec breath holds.	BHI as percentage change of CBF_v_ at end of breath hold compared to rest.	BHI was lower in both RRMS and SPMS patients compared with HC.
Sivakolundu et al. (85)	30 RRMS and 14 HC. EDSS = 2.88 ± 1.74 and disease duration = 9.60 ± 7.94 years for cognitive normal patients. EDSS = 4.4 ± 2.5 and disease duration = 17.2± 9.9 years for patients with impaired reaction time.	Patients divided into a cognitive normal group (20) and a group with reduced reaction time (10). No relapses or use of methylprednisolone within 3 months.	3T MRI. BOLD maps and CBF maps obtained concurrently by dual-echo pCASL sequence.	Inhalation of air with 5% CO_2_ for 6 min.	CVR as percentage change of CBF and BOLD signal normalized to change in P_et_CO_2_ in response to CO2 exposure.	CVR was lower in MS patients with reduced reaction time vs. normal cognition. CVR was higher in cognitively normal MS patients compared with HC.
Smoliński et al. (86)	73 RRMS patients (43 in remission and 30 with recent relapse) and 30 HC. EDSS = 1.5 (range: 0–6.0) and disease duration = 6.3 ± 4.9 years for remission patients EDSS = 4.0 (range: 2.0–4.0) and disease duration = 10.1 ± 7.3 years for patients with relapse.	Relapsing patients treated with interferon beta.	Blood velocity in MCAs (CBF_v_) by TCD.	30 sec breath holds and at least 3 min of hyperventilation.	BHI as the maximal percentage change of CBF_v_ normalized to change in PetCO_2_. HV_Δ*CO*2_ as the maximal percentage change of CBF_v_ normalized to change in PetCO_2_ during hyperventilation.	No difference in BHI or HV_Δ*CO*2_ between patients and HC. Higher BHI and HV_Δ*CO*2_ in relapsing patients after treatment with steroids.
Senzaki et al. (87)	27 MS (20 RRMS, 6 SPMS and 1 PPMS) and 24 HC. EDSS = 2.9 ± 2.6. Disease duration = 10.4 ± 8.9 years.	No relapses or use of methylprednisolone within 3 months.	Blood velocity in MCAs (CBF_v_) by TCD. FMD of the brachial artery by TCD.	30 sec breath holds for BHI. Hyperemia in brachial artery by suppression cuff for FMD.	BHI as maximal percentage change of CBF_v_ during breath hold FMD = Maximal percentage change of brachial artery diameter during hyperemia.	No difference in BHI between patients and HC. FMD reduced in patients compared with controls. FMD correlated inversely with disability scores.
Deverdun et al. (88)	35 MS (25 RRMS, 10 progressive MS, not reported whether SPMS or PPMS) and 22 HC. EDSS = 3 ± 2.1 Disease duration = 13 ± 7.6.	Same participants as in Metzger et al. (81). No relapses or use of methylprednisolone within 3 months.	3T MRI. BOLD maps measured using gradient-echo EPI sequence. White matter integrity was assessed by DTI MRI.	Inhalation of air with 8% CO_2_ for 2 min three times.	Percentage change of BOLD (CVR_BOLD_) normalized to the change in P_et_CO_2_. Only regions with reduced BOLD-signals from blood steal phenomenon during CO_2_ exposure were used in the analysis.	Patients had red- uced CVR_BOLD_ com- pared with HC in various white matter regions. Reduced CVR correlated with disease duration and verbal IQ, but not fatigue score, EDSS score or white matter integrity.

*BHI, breath hold index; BOLD, blood oxygenation level dependent; CVR_BOLD_, change in blood oxygenation level dependent from CO2 exposure; CBF, cerebral blood flow; CBF_v_, cerebral blood flow velocity; CVR, cerebrovascular reactivity; DTI, diffusion tensor imaging; EDSS, Expanded Disability Status Scale; EPI, echo planar imaging; FMD, flow mediated dilation; HC, healthy controls; HV_ΔCO2_, hyperventilation index; MCA, middle carotid arteries; MRI, magnetic resonance imaging; MS, multiple sclerosis; PCA, posterior carotid arteries; pCASL, pseudocontinuous arterial spin labeling; P_et_CO_2_, end tidal carbon dioxide partial pressure; PPMS, primary progressive multiple sclerosis; RRMS, remitting relapsing multiple sclerosis; SPMS, secondary progressive multiple sclerosis; TCD, transcranial Doppler ultrasound; TI, inversion time*.

**Table 2:** Summary of studies examining neurovascular coupling in MS.

**Paper**	**Subjects**	**Study comment**	**Modality**	**Stimulus**	**Primary outcome**	**Results**
Uzuner et al. (89)	84 MS patients (subtype not reported) and 45 HC. EDSS = 3.0 (range: 0–6.5). Disease duration = 2.9 years (range: 0.1–10.3).	Examination during exacerbation of disease.	Blood velocity in PCAs at the P2 segment (CBF_v_) by TCD.	Visual stimulation of shapes and images.	Percentage change in blood velocity (ΔCBF_v_) from visual stimulation.	MS patients had higher ΔCBF_v_ but lower resting CBF_v_ than HC.
Özkan et al. (90)	48 MS patients (subtype not reported). No HC group included. EDSS = 2.85 (range: 1–6.5). Disease duration = 2.9 years (range: 0.1–10.3).	Examination during exacerbation of disease and after 5 days of methylprednisolone treatment.	Blood velocity in PCAs at the P2 segment (CBF_v_) by TCD.	Visual stimulation by a turning cylinder which has object images on it.	Percentage change in blood velocity (ΔCBF_v_) from visual stimulation.	Intravenous high-dose methylprednisolone lowered resting CBF_v_ but did not affect ΔCBF_v_.
Reinhard et al. (91)	18 RRMS patients. No HC group included. EDSS = 2.6 ± 1.0 Disease duration = 5.6 ± 4.5 years.	Patients received regular infusion of natalizumab. Examination 4 times: 1 h before infusion; and 2 h, 2 days, and 28 days after infusion.	Blood velocity in the left PCA and right MCA (CBF_v_) by TCD.	Visual stimulation by reading a magazine.	Percentage change in blood velocity (ΔCBF_v_) from visual stimulation. The rate time as the initial steepness of CBF_v_ and the natural frequency of CBF_v_ increase was calculated.	No effect on ΔCBF_v_ but natural frequency decreased from natalizumab infusion.
Uzuner et al. (92)	389 RRMS patients and 150 HC. EDSS = 2.4 (range: 1.0–5.0). Disease duration = 2.7 years (range: 0.3–14.0).	Examination during exacerbation of disease.	TCD. Blood velocity (CBF_v_) in the left PCA and right MCA.	Visual stimulation by flickering checkerboard or by rotating optokinetic drum.	Percentage change in blood velocity (ΔCBF_v_) from visual stimulation.	ΔCBF_v_ was higher in MS patients than HC for both simple and complex visual stimulation.
Hubbard et al. (93)	28 RRMS patients and 23 HC. EDSS = 2.5 (range: 0–6). Disease duration = 12.8 ± 1.7 years.		3T MRI. BOLD maps measured by a gradient EPI sequence.	Visual stimulation by flickering checkerboard and event related paradigm by button-press task.	HRF characterized from BOLD signal. The peak amplitude, time-to-peak and FWHM of the HRF calculated as markers of NVC.	Reduced HRF amplitude peak in MS patients compared with HC.
Hubbard et al. (94)	10 MS patients (9 RRMS and 1 SPMS). No HC group included. Disease duration = 9.9 ± 1.6 [SEM] years.	No relapses or use of methylprednisolone within 1 month.	3T MRI. BOLD maps and CBF maps obtained concurrently by dual-echo pCASL sequence.	Visual stimulation by flickering white and black lines.	Percentage change in cerebral blood flow (ΔCBF) and oxygen consumption (ΔCMRO_2_) in activated areas from visual stimulation.	High ΔCBF and ΔCMRO_2_ correlated with higher fatigue score. High ΔCMRO_2_ correlated with neurological disability.
Uzuner et al. (95)	30 RRMS patients. No HC included. EDSS = 2.5 (range: 1–5.5). Disease duration = 4.8 years (range: 0.3–10.3).	Examinations during exacerbation of disease. Patients examined two times with multiple attacks in between. (0.3–10.7 years between examinations).	Blood velocity in PCAs at the P2 segment (CBF_v_) by TCD.	Visual stimulation by flickering checkerboard like pattern.	Percentage change in blood velocity (ΔCBF_v_) from visual stimulation.	ΔCBF_v_ was reduced after repeated attacks.
Stickland et al. (96)	13 RRMS patients and 10 HC. EDSS (median) = 3.0 (range: 0–4.5) Disease duration = 7.31 ± 2.06 years.		3T MRI. BOLD maps and CBF maps obtained concurrently by dual-echo pCASL sequence. Brain electrical activity measured by MEG.	Visual stimulation by flickering checkerboard like pattern.	Percentage change in cerebral blood flow (ΔCBF) in activated areas from visual stimulation. Percentage change of peak gamma power from visual activation.	ΔCBF was reduced in patients compared with HC. Electrical activity was similarly reduced.
Sivakolundu et al. (74)	37 RRMS patients and 17 HC. EDSS = 2.9 ± 1.7 and disease duration = 9.6 ± 7.9 years for speed preserved patients. EDSS = 4.4 ± 2.5 and disease duration = 17.2 ± 9.8 years for speed impaired patients.	Patients divided into cognitive speed preserved (*n* = 24) and cognitively speed impaired (*n* = 13) groups. No relapses or use of methylprednisolone within 3 months.	3T MRI. BOLD maps and CBF maps obtained concurrently by dual-echo pCASL sequence.	Visual stimulation by flickering checkerboard like pattern (8 Hz).	Percentage change in cerebral blood flow (ΔCBF) and oxygen consumption (ΔCMRO_2_) in activated areas from visual stimulation. A neurovascular coupling ratio was calculated as ΔCBF divided by ΔCMRO_2_.	No difference in ΔCBF between MS patients and HC. No difference in ΔCBF or ΔCMRO_2_ between speed impaired MS, speed preserved MS. Neurovascular ratio was reduced in speed-impaired MS patients compared with speed preserved patients.
Guo et al. (97)	56 NMO patients and 63 HC. EDSS = 3.9 (range: 0–8.5).		3T MRI. Resting state fMRI measured by gradient-echo single-short EPI sequence. CBF maps measured by pCASL sequence.	No stimulation. NVC assessed as the ratio between resting CBF and resting state fluctuations.	NVC assessed as ratio between CBF and regional homogeneity map calculated from resting state fMRI.	NVC was reduced in parietal and occipital regions but increased in temporal and pre- frontal regions compared with HC. Changes in patients correlated with disease severity and cognitive deficits.
Turner et al. (98)	30 RRMS patients and 25 HC. EDSS = 2.8 (SEM = 0.4) Disease duration = 12.8 years (SEM = 1.2).		3T MRI. BOLD maps measured by gradient-echo EPI sequence.	Visual stimulation by flickering checkerboard like pattern and cognitive stimulation by digit symbol substitution test.	HRF characterized from BOLD signal. The peak amplitude, time-to-peak and FWHM of the HRF calculated as markers of NVC.	Peak amplitude was reduced, and time-to-peak was longer in MS patients compared with HC. Faster time-to-peak correlated with processing speed in patients.

*BHI, breath hold index; BOLD, blood oxygenation level dependent; cBOLD, calibrated BOLD; CBF, cerebral blood flow; ΔCBF, change in cerebral blood flow from neuroactivation; CBF_v_, cerebral blood flow velocity; ΔCBF_v_, change in cerebral blood flow velocity from neuroactivation; CMRO_2_, cerebral metabolic rate of oxygen; ΔCMRO_2_, change in cerebral metabolic rate of oxygen from neuroactivation; EDSS, Expanded Disability Status Scale; EPI, echo planar imaging; FWHM, full width half maximum; HC, healthy controls; HRF, hemodynamic response function; MCA, middle carotid arteries; MEG, magnetoencephalography; MRI, magnetic resonance imaging; MS, Multiple Sclerosis; NVC, neurovascular coupling; PCA, posterior carotid arteries; pCASL, pseudocontinuous arterial spin labeling; PPMS, primary progressive multiple sclerosis; RRMS, remitting relapsing multiple sclerosis; SEM, standard error of the mean; SPMS, secondary progressive multiple sclerosis; TCD, transcranial Doppler ultrasound*.

## Results

### Literature Search

**Figure 1:**
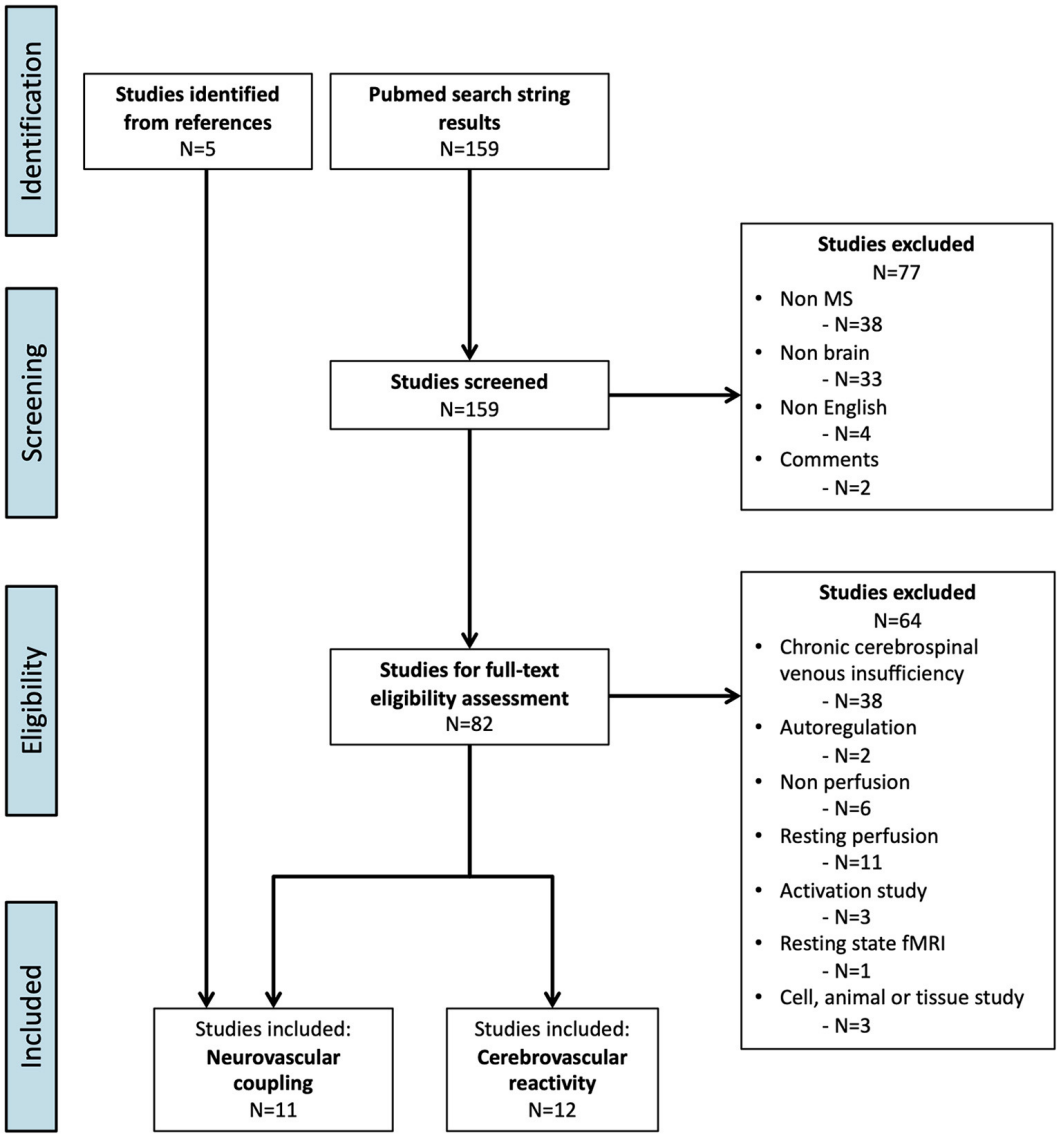
Flowchart of the inclusion process (PRISMA flow diagram) (76).

The PubMed search resulted in 159 articles. Twelve articles examined CVR (Figure 1; Table 1). Six articles examined NVC and five additional studies were included from the reference lists, resulting in a total of 11 articles on NVC (Figure 1; Table 2).

### Cerebrovascular Reactivity

A summary of the 12 studies on CVR is presented in Table 1. All studies included a healthy control (HC) group for comparison with the MS patient group. The studies demonstrated mixed results. Six studies resulted in no differences between MS patients and HC, and 5 studies resulted in reduced CVR in patients compared with HC. In one study, the controls consisted of migraine patients, where MS patients demonstrated higher CVR than migraine patients. All studies used CO_2_ as the vasodilating agent, either by inhaling air with added CO_2_ or through breath-hold challenges. The studies used either the TCD methodology (*n* = 6), the BOLD imaging MRI technique (*n* = 2) or the ASL MRI technique (*n* = 4). The participating patients were primarily relapsing remitting MS (RRMS) patients; however, in some studies, patients with secondary progressive MS (SPMS) were also included.

### Neurovascular Coupling

A summary of the 11 studies on NVC is presented in Table 2. In eight of the studies, HC were included for comparison. These studies showed mixed results: two studies demonstrated higher NVC in MS patients; two studies found no differences; three studies reported reduced NVC in patients; and one study observed increased NVC in some brain regions but decreased NVC in other brain regions compared with HC. Four of the studies used TCD Doppler to measure the increase in blood velocity in the PCA or MCA from visual stimulation as a marker of NVC. Three of the studies used calibrated BOLD and ASL MRI to measure the increase in CBF and CMRO_2_ in response to visual stimulation in the visual cortex. One of the studies used a combination of resting-state fMRI and ASL to acquire metrics describing NVC. Two studies used conventional BOLD imaging and calculated the characteristics of the HRF as markers of NVC.

All studies used visual stimulation for neuronal activation, except for Hubbard et al. (93), who used a combination of visual stimulation and a button press task, and Turner et al. (98), who used a combination of visual stimulation and a cognitive task.

The patients included in the studies consisted of RRMS patients, except for the work of Hubbard et al. (94), in which the studied population consisted of RRMS and one SPMS patient, and that of Guo et al. (97) who examined neuromyelitis optica (NMO) patients.

## Discussion

### Cerebrovascular Reactivity

In all studies, CO_2_ was used as the vasodilating agent, either by inhalation of air with added CO_2_ or through breath-hold challenges. The studies found either no difference or a reduced CVR between MS patients and HC.

Using the ASL MRI technique, Marshall et al. demonstrated in 2014 (78), and again in 2016 (79), that patients with MS have a reduced CVR in response to the inhalation of air with 5% CO_2_ compared with HC. The mean Expanded Disability Status Scale (EDSS) score was 2.9, and most subjects received second-line treatment. Recent relapse was not an exclusion criterion. A more recent study by Pelizzari et al. (83) that also used ASL MRI found no significant differences in CVR between MS patients and HC from inhalation of air with 5% CO_2_. This cohort had a mean EDSS of 1.5 and predominantly received no treatment or first-line treatment. Patients were excluded from the study if they had experienced a recent relapse. The authors speculate that the low degree of inflammation in their MS cohort may explain the lack of difference between patients and HC (83).

The role of severity and disease stage in the magnitude of possible CVR changes in MS was suggested in a recent study by Lattanzi et al. (84), who used TCD to measure the breath-hold-index (BHI) as a marker of CVR in a total of 80 RRMS patients outside of relapse and 80 HC, making it the largest current TCD study addressing CVR differences in MS. The authors report a groupwise progressively decreasing BHI from HC to RRMS and SPMS. The authors used bilateral simultaneous measurements of the MCA, repeating the breath hold experiment three times for each subject, and then calculating their mean value. This strategy reduced the variability of the reported BHI when compared with other TCD studies. Smoliński et al. (86), who found no difference in BHI between MS patients and HC, performed one unilateral measurement in each subject and reported a standard deviation in RRMS patients outside of relapse 2.5 times larger than the reported results of Lattanzi et al. (84) This finding indicates that for BHI, which is a method reported to have a good short-term but poor long-term reproducibility, a concern is methodological variability.

Several smaller TCD studies have also reported no difference in BHI between MS patients and HC; however, they applied variable methodologies, which ranged from using a short breath hold of 15 sec (77), which may not be long enough to achieve a sufficient CO_2_ increase (99), to using migraine patients as control subjects (80). It is noteworthy that most of these negative studies have utilized a single unilateral measurement and generally reported relatively high standard deviations of the measured BHI (86, 87). Finally, three of these negative studies showed a non-significant trend toward lower BHI in RRMS when compared with HC (77, 82, 87), and may have been underpowered and unable to detect potentially small differences. Two studies utilized the change in MRI BOLD response to CO_2_ inhalation as a marker of CVR and reported that CVR changes may be secondary to cognitive impairment in MS patients (81, 85). However, a similar relationship has been observed for neurodegenerative diseases (100), and thus, it remains to be seen how much of the CVR changes observed in MS are driven by early cognitive impairment, since this potential confounder has not been addressed in most studies. Other potential confounding factors may be recent relapses and steroid treatment, which has been found to significantly decrease CVR on a single subject level in one study (86).

#### Methodological Considerations

Burley et al. (101) investigated the correlations between four different methods for estimating CVR, namely TCD, BOLD MRI, ASL MRI and PCM MRI, in healthy subjects (101). The authors found no clear relationship between TCD and BOLD measures, highlighting that the different methodological approaches address different anatomical parts of the vasculature (TCD: arteries, BOLD: venules/veins, ASL: arterioles and capillaries). This finding calls for caution when comparing the CVR metrics derived from different imaging modalities. On the positive side, resting CBF was highly correlated between ASL, PCM and TCD, suggesting that vasodilation by CO_2_ cannot automatically be assumed to exert similar homogenous effects throughout the different anatomical parts of the cerebral vasculature.

The TCD methodology has an inherent problem when attempting to address changes in blood flow and thus CVR, because it does not directly measure flow but rather velocity. To use the blood velocity as a proxy for blood flow, the assumption that there is no change in the cross-sectional area of the artery following a stimulus must be valid. If such an effect differs significantly between the groups to be compared, for example between HC and patients, true physiological differences may be overlooked. Recently, it has been pointed out that blood vessel diameter is likely to be affected in the context of a gas challenge (102, 103), coinciding with an increase in blood velocity. This factor leads to an overestimation of CVR from a gas challenge in cases where blood vessels are less pliable.

### Neurovascular Coupling

Studies examining NVC demonstrate mixed and contradictory results. Three studies, Stickland et al. (96), Sivakolundu et al. (74) and Hubbard et al. (94) measured the change in CBF from neuronal activation (ΔCBF) in the visual cortex using the ASL MRI technique. Furthermore, Hubbard et al. (94) and Sivakolundu et al. (74) additionally calculated the change in CMRO_2_ (ΔCMRO_2_) using a calibrated BOLD technique to examine the coupling between CBF and CMRO_2_ as a marker for NVC. Stickland et al. (96) acquired brain electrical activity using MEG to examine the NVC as the coupling between CBF and MEG gamma band power. Guo et al. (97) also examined NVC using the BOLD and ASL techniques. However, the study did not measure NVC in relation to external stimuli but examined the NVC from the resting fluctuation of brain activity and CBF in the resting state networks.

Sivakolundu et al. (74) found no differences in the ΔCBF response to visual stimulation between MS patients and HC. The study further examined the differences in ΔCBF response between MS patients with reduced cognitive processing speed and patients with normal processing speed but no significance was observed. However, when examining the ratio between ΔCBF and ΔCMRO_2_ as a marker of NVC, they found the ratio to be significantly reduced in patients with impaired processing speed, suggesting a neurovascular uncoupling. The authors hypothesize that the impaired NVC can, in part, explain the reduced processing speed and cognitive impairment observed in some MS patients. Stickland et al. (96) observed a significantly smaller ΔCBF in MS patients than in HC. However, the neuronal response measured by the MEG gamma band power was similarly reduced, which indicates that the NVC is not affected in MS. This finding is in contrast to the results of Sivakolundu et al. (74) who observed no differences in ΔCBF but found a reduction of NVC in MS. In contrast to both the studies of Stickland et al. (96) and Sivakolundu et al. (74), Hubbard et al. (94) found that a higher ΔCBF and ΔCMRO2 was correlated with fatigue and more severe MS disease, suggesting that NVC was increased in MS patients. The higher metabolic response was also correlated with white matter myelin abnormalities examined by the diffusion tensor imaging (DTI) MRI technique. The study did not include a control group and the group of patients was small (*n* = 10). Finally, Guo et al. (97) examined NVC in NMO patients based on the resting state fluctuation of the BOLD signal and CBF in the brain using fMRI and ASL MRI techniques. The study found that NVC was reduced in some brain regions but increased in other regions in NMO patients compared with HC. The different NVCs, both higher and reduced NVC, correlated with the disease severity of the NMO patients.

Two studies by Hubbard et al. (93) and Turner et al. (98) indirectly examined the CBF response to activation using traditional BOLD imaging, in which the peak of amplitude and the time to peak of the BOLD HRF were used to describe the NVC. Both studies found that the peak amplitude of the BOLD signal was reduced in MS patients compared with HC, suggesting a reduced cerebrovascular response in MS. Turner et al. (98) also demonstrated a longer time to peak in MS patients and that a longer time to peak correlated with poor cognitive processing speed performance, indicating a correlation between cognitive decline and an impaired CBF response to neuroactivation.

Two studies by Uzuner et al. (89, 92) indirectly examined the CBF response to neuroactivation by measuring the blood velocity in PCA (ΔCBF_v_) using TCD. Both studies found higher CBF_v_ from visual stimulation in patients with MS during a period of exacerbation of the disease compared with HC. The studies also demonstrated a lower resting CBF_v_ in MS patients than in HC. Overall, these results correspond with Hubbard et al. (93), who found higher ΔCBF in MS patients measured using the ASL MRI technique.

Two studies examined whether infusion of MS medication affected NVC. Özkan et al. (90) and Reinhard et al. (91) examined NVC before and after infusion of methylprednisolone or natalizumab, respectively. Both studies used TCD to examine the NVC. Methylprednisolone treatment lowered the resting CBF_v_; however, ΔCBF_v_ from neuroactivation was unaffected. Treatment with natalizumab did not affect ΔCBF_v_ but increased the very initial response in ΔCBF_v_ from neuroactivation. Furthermore, the authors suggest that these findings are evidence for increased reactivity of the neurons or cerebral vessels after treatment.

The cause for these mixed observations is not obvious. Potential causes for differences in ΔCBF and ΔCMRO_2_ could be due to the heterogeneity of the examined patients with important differences in factors such as age, disease severity, MS subtype and treatment status. Variation in the degree of atrophy in MS patients could also affect the metabolic response to activation, as patients with MS demonstrate increased brain atrophy compared with age-matched control subjects (104–107). If the examined MS patients have fewer neurons due to atrophy, the metabolic response to activation would also be reduced. Ideally, studies should acquire some measure of atrophy or cortical thickness to address this potential confounder. However, brain volume estimation in MS patients must be interpreted with caution since MS patients can exhibit transient increases in brain volume due to inflammatory processes causing increased water and inflammatory cell volume. This phenomenon is often referred to as pseudoatrophy, as it is often observed in MS patients following disease-modifying anti-inflammatory treatment (104, 108). Therefore, differences in brain volume therefore do not necessarily directly reflect changes in neurons or glia cells. For example, Stickland et al. (96), who observed a reduced CBF response in MS patients, examined the gray matter volume in the visual cortex and did not observe a higher degree of atrophy in the MS patients than in the HC. This indicates that the reduced metabolic response is not a result of fewer viable neurons. However, the authors speculate that the lack of differences in atrophy between patients and HC could be due to the inflammatory processes in the MS brain causing swelling of the brain, resulting in higher brain volume estimates derived from anatomical images.

Some studies have demonstrated higher ΔCBF or NVC in MS patients. Hubbard et al. (94), who observed that higher ΔCBF and ΔCMRO_2_ were correlated with more severe MS disease, suggests that the reason for a higher metabolic response in MS could be due to a reduced resting metabolism. If the patients have reduced resting metabolism, they will demonstrate a higher percentage increase in CBF and CMRO_2_ in response to neuroactivation. The study did not include a control group to test this hypothesis. However, other studies have demonstrated reduced resting perfusion in MS patients that correlates with disease severity (32, 109, 110). A study by West et al. (34) have subsequently demonstrated that MS patients also have lower resting CMRO_2_ than HC. However, in this study, it was also observed that patients with high resting CMRO_2_ had worse fatigue and cognitive symptoms, which contrasts with the hypothesis of higher NVC due to lower resting metabolism in patients with more severe disease symptoms.

Uzuner et al. (89, 92) found that ΔCBF_v_ was higher in MS patients who were examined during disease exacerbation. The authors suggest that patients with MS have more reactive neurons and cerebral vessels in response to neuroactivation during active disease. A subsequent study from Uzuner et al. (95), where patients were examined again after 26.7 months (range: 4–120 months) of disease progression, demonstrated that ΔCBF_v_ was reduced after repeated relapses. The authors suggest that following disease progression, neuronal damage secondary to inflammatory activity inhibits the neurovascular unit and reduces NVC, which suggests that MS disease will progressively impair NVC over time.

#### Methodological Considerations

The use of ASL to measure increases in CBF from neuroactivation has good reproducibility (68) and has been validated against ^15^O-H_2_O PET imaging (65), which is considered the best available method for the quantification of cerebral perfusion. However, CBF measurements from different ASL MRI techniques differ significantly between MRI scanners and the applied data acquisition sequence. Thus, the heterogeneity of the ASL sequences and applied methodology between research groups could potentially bias the results (111, 112).

The examination of NVC by TCD is an indirect measurement because it measures the change in blood velocity in a large upstream artery supplying the activated region of the brain. Several TCD studies have investigated the magnitude of the increase in blood velocity from neuroactivation by a visual stimulation as well as a cognitive task. However, to our knowledge, whether the percentage increase in blood velocity measured by TCD is proportional to the actual NVC in the affected tissue has not yet been validated. The indirect measurement of the upstream effect will have confounding factors. For instance, most notably, the effect of increased perfusion in the activated region will be greatly diluted as the large artery also supplies other regions of the brain. Therefore, a clear confounding factor of this upstream measurement is therefore the size of the activated region, as larger activated areas will have stronger effects on the measurement compared with smaller activated areas. Thus, when examining the NVC, the primary interest is not to measure the size of the activated area but instead to measure how much CBF has increased in the activated region. This factor makes it challenging to draw a conclusion about the amplitude of NVC when measuring the change in upstream blood flow velocity by TCD. Therefore, direct comparisons between studies using TDCs and studies using other neuroimaging techniques to examine cerebrovascular responses to neuroactivation should be performed with a high degree of caution.

The larger ΔCBF_v_ observed in MS patients can also be a result of a failure to fulfill the assumption that there is no significant difference exists in the cross-sectional area of the artery following stimulation between the studied groups. The MS patients may demonstrate a smaller change in the cross-sectional area, which increases blood flow velocities to ensure an increase in CBF from activation. Similar concerns are present when examining CVR from CO_2_ stimulation as discussed earlier. Senzaki et al. (87) have demonstrated that MS patients have reduced dilation of the brachial artery from reactive hyperemia, suggesting an inhibited vasodilation in MS patients, perhaps due to endothelial dysfunction.

#### Activation Pattern Studies

Several studies have examined neuroactivation in MS using BOLD imaging and have found alterations in activation patterns (113–120). Most studies have shown larger activation patterns in MS, which could be a result of a hyperreactivity of neurons and cerebral vessels, resulting in a higher NVC in MS patients. The BOLD signal is generated by the NVC, and changes in this coupling would likely cause alterations of the activation patterns seen in MS patients.

## Conclusion

Due to increasing evidence for tissue hypoxia and endothelial vessel damage in MS, it seems plausible to speculate that MS patients exhibit impaired cerebrovascular reactivity and perfusion modulation. Reduced cerebrovascular function is associated with declining brain health, especially in the context of age-related neurodegeneration. Patients with MS demonstrate a progressive decline in brain health in the form of neurodegeneration with advancing disease stages. Whether these processes are, at least partially, related to cerebrovascular function warrants a thorough investigation.

Studies on CVR in MS patients demonstrate either no significant differences between patients and HC or reduced CVR in patients. This discrepancy can be because CVR is not affected in early MS disease but becomes progressively more affected after a certain disease period, with advancing disease stage.

The studies on NVC demonstrate diverse results, and a conclusion that describes all the published observations is difficult to establish. Some studies found indications of an increased CBF response to neuroactivation and increased NVC, especially during exacerbation of disease, perhaps due to an increase in reactive neurons. A higher NVC can also be a result of a lower resting metabolism, which then causes a larger percentage increase in CBF and CMRO_2_ in response to neuronal activation. Other studies found indications of a reduced CBF response to activation, which can be a result of impaired vasodilation and endothelial function in MS patients. More studies are needed to elaborate on these findings. Ideally, future studies should directly measure the increase in CBF and neuronal activity. A measure of brain atrophy should be included to address the role of atrophy in explaining the changes in NVC. Resting perfusion and metabolism should also be acquired to investigate whether reduced resting physiology can explain a relatively higher cerebrovascular and neuronal activity response in MS patients. Finally, the studies should have sufficient power to examine the effect of confounding factors such as age, MS subgroup, disease severity, treatment status and recent acute inflammatory events.

## Data Availability Statement

The original contributions presented in the study are included in the article/supplementary materials, further inquiries can be directed to the corresponding author.

## Author Contributions

MV and HL conceived and designed the study. MV and SC collected the data and wrote the paper. JF and HL edited and revised the manuscript. All authors contributed to the article and approved the submitted version.

## Funding

MV was funded by grant from the Lundbeck Foundation (R347-2020-217).

## Conflict of Interest

The authors declare that the research was conducted in the absence of any commercial or financial relationships that could be construed as a potential conflict of interest.

## Publisher's Note

All claims expressed in this article are solely those of the authors and do not necessarily represent those of their affiliated organizations, or those of the publisher, the editors and the reviewers. Any product that may be evaluated in this article, or claim that may be made by its manufacturer, is not guaranteed or endorsed by the publisher.
